# Adoptive Cell Transfer of Regulatory T Cells Exacerbates Hepatic Steatosis in High-Fat High-Fructose Diet-Fed Mice

**DOI:** 10.3389/fimmu.2020.01711

**Published:** 2020-07-31

**Authors:** Mikhaïl A. Van Herck, Luisa Vonghia, Wilhelmus J. Kwanten, Thomas Vanwolleghem, Didier G. Ebo, Peter P. Michielsen, Joris G. De Man, Lucio Gama, Benedicte Y. De Winter, Sven M. Francque

**Affiliations:** ^1^Translational Research in Immunology and Inflammation, Laboratory of Experimental Medicine and Pediatrics, Division of Gastroenterology and Hepatology, University of Antwerp, Antwerp, Belgium; ^2^Department of Gastroenterology and Hepatology, Antwerp University Hospital, Edegem, Belgium; ^3^Infla-Med Centre of Excellence, University of Antwerp, Antwerp, Belgium; ^4^Translational Research in Immunology and Inflammation, Immunology-Allergology-Rheumatology, University of Antwerp, Antwerp University Hospital, Antwerp, Belgium; ^5^Department of Molecular and Comparative Pathobiology, Johns Hopkins School of Medicine, Baltimore, MD, United States

**Keywords:** non-alcoholic steatohepatitis, adipose tissue inflammation, adoptive cell transfer, regulatory T cells, T helper 1 cells, PPAR-γ

## Abstract

**Background and Aims:** Non-alcoholic steatohepatitis (NASH) is a multisystem condition, involving the liver, adipose tissue, and immune system. Regulatory T (Treg) cells are a subset of T cells that exert an immune-controlling effect. Previously, a reduction of Treg cells in the visceral adipose tissue (VAT) was shown to be associated with a more severe degree of liver disease. We aimed to correct this immune disruption through adoptive cell transfer (ACT) of Treg cells.

**Methods:** Male 8-week-old C57BL/6J mice were fed a high-fat high-fructose diet (HFHFD) for 20 weeks. Treg cells were isolated from the spleens of healthy 8 to 10-week-old C57BL/6J mice and were adoptively transferred to HFHFD-fed mice. PBS-injected mice served as controls. Plasma ALT and lipid levels were determined. Liver and adipose tissue were assessed histologically. Cytotoxic T (Tc), Treg, T helper (Th) 1 and Th17 cells were characterized in VAT, liver, subcutaneous adipose tissue (SAT), blood, and spleen via flow cytometry. Gene expression analysis was performed in SAT and VAT of mice fed either the HFHFD or a control diet for 10–32 weeks.

**Results:** ACT increased Treg cells in SAT, but not in any of the other tissues. Moreover, the ACT induced a decrease in Th1 cells in SAT, liver, blood, and spleen. Higher plasma ALT levels and a higher degree of steatosis were observed in ACT mice, whereas the other HFHFD-induced metabolic and histologic disruptions were unaffected. Expression analysis of genes related to Treg-cell proliferation revealed a HFHFD-induced decrease in all investigated genes in the SAT, while in the VAT the expression of these genes was largely unaffected, except for a decrease in *Pparg*.

**Conclusion:** ACT of Treg cells in HFHFD-fed mice exacerbated hepatic steatosis, which was possibly related to the increase of Treg cells in the SAT and/or the general decrease in Th1 cells. Moreover, the HFHFD-induced decrease in *Pparg* expression appeared critical in the decrease of Treg cells at the level of the VAT and the inability to replenish the amount of Treg cells by the ACT, while the mechanism of Treg cell accumulation at the level of the SAT remained unclear.

## Introduction

Non-alcoholic fatty liver disease (NAFLD) is defined by the presence of hepatic steatosis and comprises a spectrum of gradually progressive disease states. The more active form, non-alcoholic steatohepatitis (NASH) is characterized histologically by the presence of both hepatocellular ballooning—a sign of cell damage—and lobular inflammation. NASH is associated to several comorbidities and may progress to increasing degrees of liver fibrosis, eventually leading to liver cirrhosis with all its associated complications, which underlines the unmet need for an efficacious medical treatment (1, 2). As a very strong association exists between NAFLD and the metabolic syndrome—clustering visceral overweight, dyslipidemia, insulin resistance, and arterial hypertension—it has been postulated that the former is actually the hepatic manifestation of the latter (1, 2). Furthermore, it has been shown that NAFLD is no mere hepatic disease, as a strong interaction exists—both clinically and pathophysiologically—between the liver and other organ systems, including the gut microbiome, cardiovascular system, and adipose tissue. The pathogenesis of NASH is a complex and multifactorial process, comprising multiple parallel hits. Previous research has shown that both the innate and adaptive immune systems play an important role in NASH development and progression. However, the exact actors and mechanisms remain to be fully elucidated (3, 4).

Regulatory T (Treg) cells are defined by the expression of the transcription factor forkhead box P3 (Foxp3) and exert an immune-controlling effect. Their main action is to prevent autoreactivity toward self-antigens and to avoid excessive effector-T-cell activation and subsequent tissue damage during infection-induced immune responses and inflammation (4). It has been demonstrated that Treg cells are highly enriched in the visceral adipose tissue (VAT), but not in the subcutaneous adipose tissue (SAT), of lean mice and that they possess a transcriptionally unique phenotype compared to Treg cells in peripheral lymphoid tissues (5, 6). We and others previously demonstrated a reduction of Treg cells in the VAT in diet-induced obesity in mice (6–9), which was associated with a higher degree of insulin resistance (6) and NAFLD (9). To investigate the mechanistic involvement of adipose tissue Treg cells in the pathogenesis of NASH, we aimed to correct the reported immune disruption at the level of the VAT through an adoptive cell transfer (ACT) of Treg cells, entailing the transfer of cells from donor mice to acceptor mice. This technique has previously been proven a valuable gain-of-function method to explore the mechanistic involvement of Treg cells (10, 11).

## Materials and Methods

### Mice

Eight-week-old C57BL/6J mice were purchased at Janvier Labs (Le Genest-Saint-Isle, France). They were kept in a 12 h:12 h light/dark cycle with controlled temperature and humidity and were housed in enriched (wooden rods and polycarbonate house) cages with a stainless-steel grid and filter top in accordance to established guidelines. All interventions were performed during the light cycle. Depending on the experimental group, mice had free access to standard chow (Carfil Quality, Oud-Turnhout, Belgium) which served as a control diet (CD) or a high-fat high-fructose diet (HFHFD, D16042610, Research Diets, New Brunswick, NJ, USA), containing 55 kcal% fat and 14 kcal% fructose. This study was approved by the Ethical Committee for Animal Testing, University of Antwerp (Antwerp, Belgium), ID 2015-16. Lab animal-specific information was reported in accordance with the ARRIVE guidelines.

### Adoptive Cell Transfer

CD4^+^ CD25^+^ regulatory T cells were isolated from the spleens of healthy 8 to 10-week-old mice with the CD4^+^ CD25^+^ Regulatory T Cell Isolation Kit (Miltenyi Biotec, Bergisch Gladbach, Germany). The purity of the isolated cells was evaluated by means of flow cytometry. They consisted out of CD4^+^ CD25^+^ Foxp3^+^ cells for 96%. Subsequently, mice that were fed the HFHFD for 20 weeks (*N* = 8) were injected intraperitoneally with either ~1 × 10^6^ isolated Treg cells suspended in 500 μL of phosphate-buffered saline (PBS, Thermo Fisher Scientific, Waltham, MA, USA) at a ratio of 2–3 donor mice to 1 acceptor mouse, or an equal volume of PBS (*N* = 7) according to an established protocol (12, 13). Mice were sacrificed 3 days after the procedure, aiming to permit the transferred Treg cells a sufficient amount of time to elicit their anti-inflammatory effect, while accounting for the estimated duration of their *in vivo* survival (14).

### Procurement of Target Tissues, Immune Cell Isolation and Flow Cytometry

**Figure 1:**
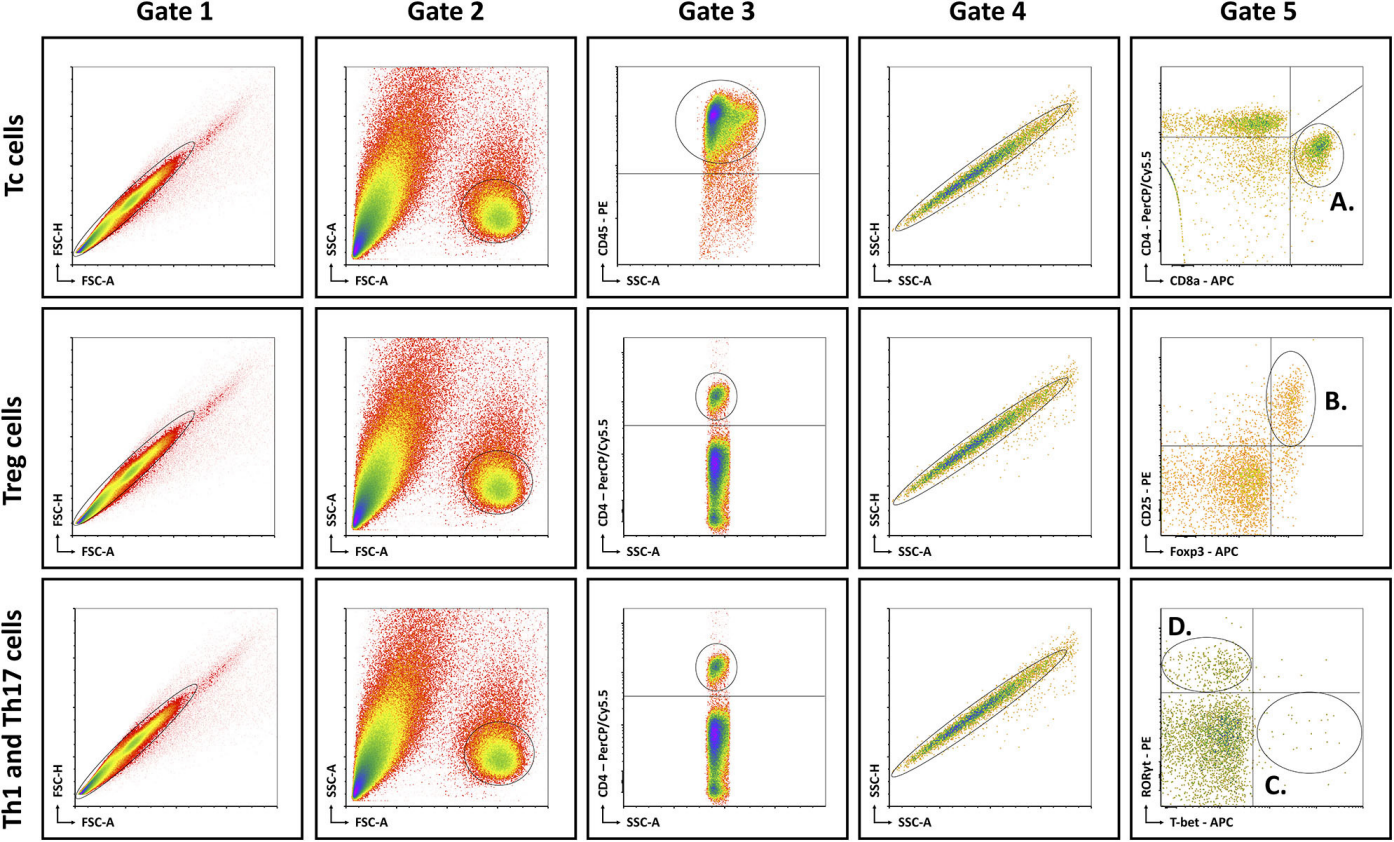
Schematic overview of flow cytometric gating strategy for the identification of **(A)** Tc cells, **(B)** Treg cells, **(C)** Th1 cells, and **(D)** Th17 cells in liver, VAT, SAT, spleen, and blood. Lymphocyte populations were determined by FSC-A and SSC-A. Doublets and triplets were filtered out twice by plotting area to height for both FSC and SSC. Concerning the fluorochrome-stained cells, positive populations were defined as being above the 99th percentile of FMO staining, indicated by the quadrants. -A, area; FSC, forward scatter; FMO, fluorescence minus one; -H; height; SSC, side scatter; Tc, cytotoxic T; Th1, T helper 1; Th17, T helper 17; Treg, regulatory T.

Mice were anesthetized with pentobarbital (0.12 mg per gram of bodyweight, Nembutal, Ceva, Libourne, France) at 8–9 am, without being fasted. Blood was collected on EDTA by cardiac puncture. Liver, visceral adipose tissue (VAT), subcutaneous adipose tissue (SAT), and spleen were excised and processed as described previously (15). The resulting single cell suspensions were incubated at 4°C at approximately 1 × 10^6^ cells per 100 μL with fluorochrome-conjugated antibodies (Table 1). Flow cytometry was performed on an Accuri C6 (BD Biosciences, Franklin Lakes, NJ, USA). Data analysis was performed with FCS Express 4 (De Novo Software, Glendale, CA, USA). Lymphocyte populations were determined by forward scatter and side scatter. Doublets and triplets were filtered out twice by plotting area to height for both forward scatter and side scatter. Concerning the fluorochrome-stained cells, positive populations were defined as being above the 99th percentile of fluorescence-minus-one (FMO) staining. The gating strategy is depicted in Figure 1.

**Table 1:** Overview of antibodies used in flow cytometry.

	**Antigen**	**Fluorochrome**	**Clone**	**Supplier**
Flow cytometry	CD45	PE	30-F11	BioLegend
Flow cytometry	CD4	PerCP/Cy5.5	GK15	BioLegend
Flow cytometry	CD8a	APC	53-6.7	BioLegend
Flow cytometry	CD25	PE	PC61	BioLegend
Flow cytometry	Foxp3	APC	FJK-16s	Thermo Fisher Scientific
Flow cytometry	T-bet	APC	4B10	Thermo Fisher Scientific
Flow cytometry	RORγt	PE	B2D	Thermo Fisher Scientific

### Biochemical Analysis

Whole blood samples were centrifuged for 10 min at 1,500 G. Plasma was stored at −20°C for a maximum of 14 days. Biochemical analysis was performed with an automated Dimension Vista 1500 System (Siemens Healthineers, Erlangen, Germany) and included aspartate aminotransferase (AST), alanine aminotransferase (ALT), total cholesterol, high-density lipoprotein (HDL), low-density lipoprotein (LDL), and triglycerides.

### Histology

Liver, VAT, and SAT were prepared for histological analysis and stained according to standard lab protocol with hematoxylin-eosin (H&E), Masson's trichrome (MT), and Oil-red-O as previously described (16). Immunohistochemistry was performed using the anti-CD45 antibodies (ab10558, Abcam, Cambridge, UK), a general leukocyte marker. Secondary antibodies were species-appropriate horseradish peroxidase conjugates (Vectastain ABC; Vector Laboratories, Burlingame, CA, USA).

The histological hallmarks of NASH in the liver were assessed on H&E-stained slides by means of the NAFLD Activity Score (NAS) (17). Steatosis was quantified on Oil-red-O-stained slides with ImageJ (National Institutes of Health, Bethesda, MD, USA) and expressed as percentage stained. Hepatic inflammation was quantified immunohistochemically by quantification of the amount of CD45^+^ inflammatory foci per mm^2^ (ImageJ). Fibrosis was graded according to the accepted NASH-CRN definitions (17) and quantified with ImageJ by measuring the stained area, both on MT-stained slides. Adipocyte diameters in VAT and SAT were determined with the ImageJ plugin Adiposoft (18) and adipose tissue inflammation was quantified immunohistochemically as the number of CD45^+^ inflammatory foci per mm^2^ with ImageJ.

### Gene Expression Analysis

Mice were fed either the HFHFD or the CD and sacrificed at 10-15-20-25-32 weeks after the start of the experiment (*N* = 6–12 per time point, per group). Based on their similar phenotype, mice were pooled as follows: 10–15, 20–25, and 32 weeks (9). At sacrifice, SAT and VAT were snap-frozen in liquid nitrogen and stored at −80°C. A random selection was made of 5–6 samples from CD-fed and HFHFD-fed mice at every grouped time point (10–15, 20–25, and 32 weeks). Subsequently, the tissues were homogenized (Precellys Lysing Kit, Bertin Instruments, Montigny-le-Bretonneux, France) and total RNA was extracted from SAT and VAT (RNeasy Lipid Tissue Mini Kit, Qiagen, Hilden, Germany). RNA expression of 561 genes was determined (nCounter Mouse Immunology panel, NanoString Technologies, Seattle, USA). Following hybridization, transcripts were quantified (nCounter, NanoString Technologies). Results were normalized in the nSolver Analysis Software (NanoString Technologies) by the geometric mean of 14 housekeeping genes and six positive controls. *Pparg, Il33, Il1rl1, Stat6, Il10, Tgfb1, Stat5a*, and *Il2* expression was quantified as a part of the nCounter Immunology panel.

### Statistical Analysis

For statistical analysis, the Mann Whitney U-test or Wilcoxon signed rank test was used as appropriate in SPSS 25 (IBM, Armonk, NY, USA). Significance was assumed at *p* < 0.05. Graphs were designed in Graphpad Prism 7 (GraphPad Software, San Diego, CA, USA). Group size was 7–8 for the ACT experiment and 5–6 for the gene expression experiment.

## Results

**Figure 2:**
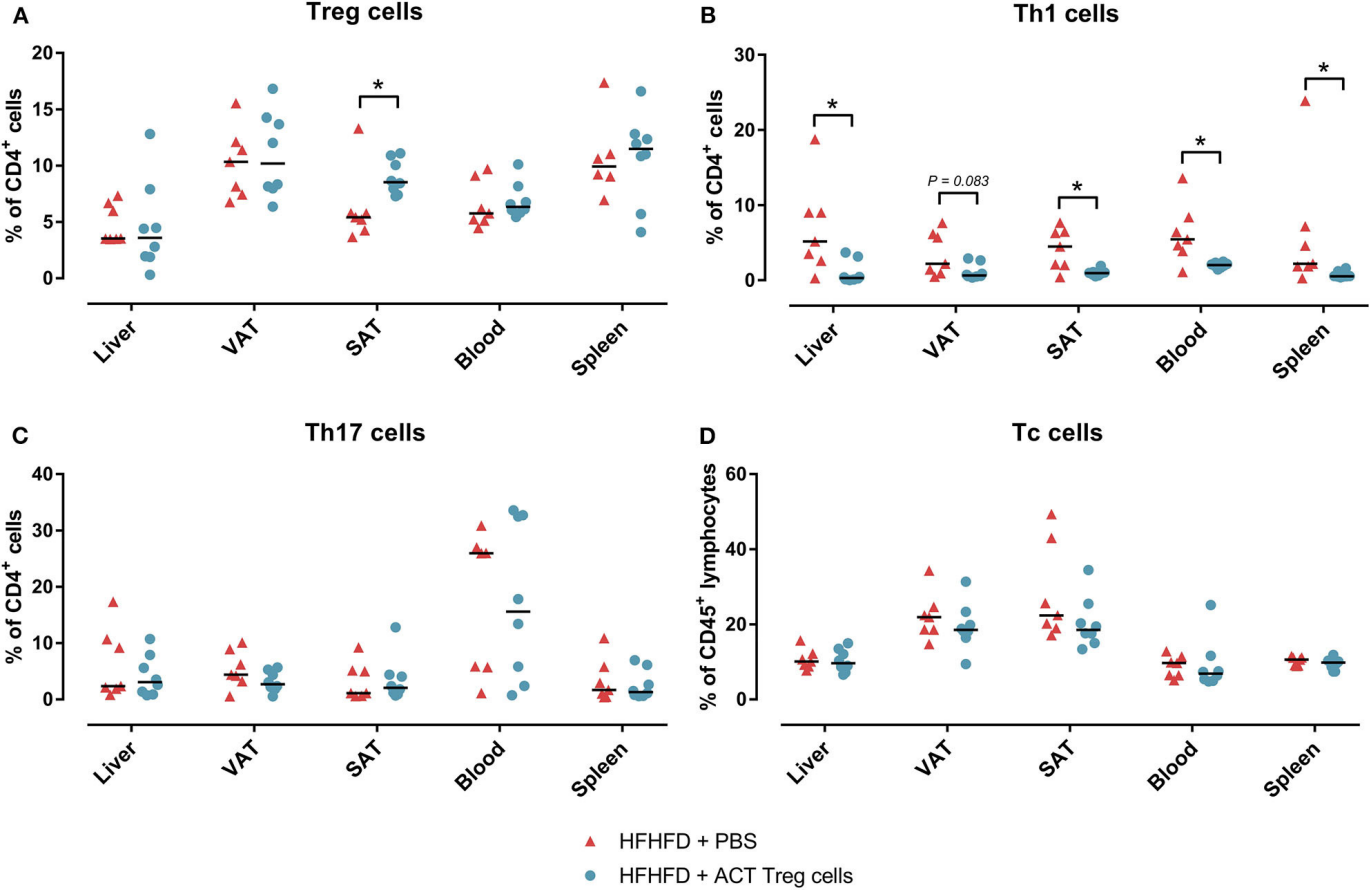
The effect of adoptive Treg cell transfer on **(A)** Treg, **(B)** Th1, **(C)** Th17, and **(D)** Tc cell numbers in liver, VAT, SAT, spleen, and blood. Medians are depicted with a horizontal black line. *P < 0.05 (Mann Whitney U). ACT, adoptive cell transfer; PBS, phosphate-buffered saline; SAT, Tc, cytotoxic T; Th1, T helper 1; Th17, T helper 17; SAT, subcutaneous adipose tissue; VAT, visceral adipose tissue.

**Figure 3:**
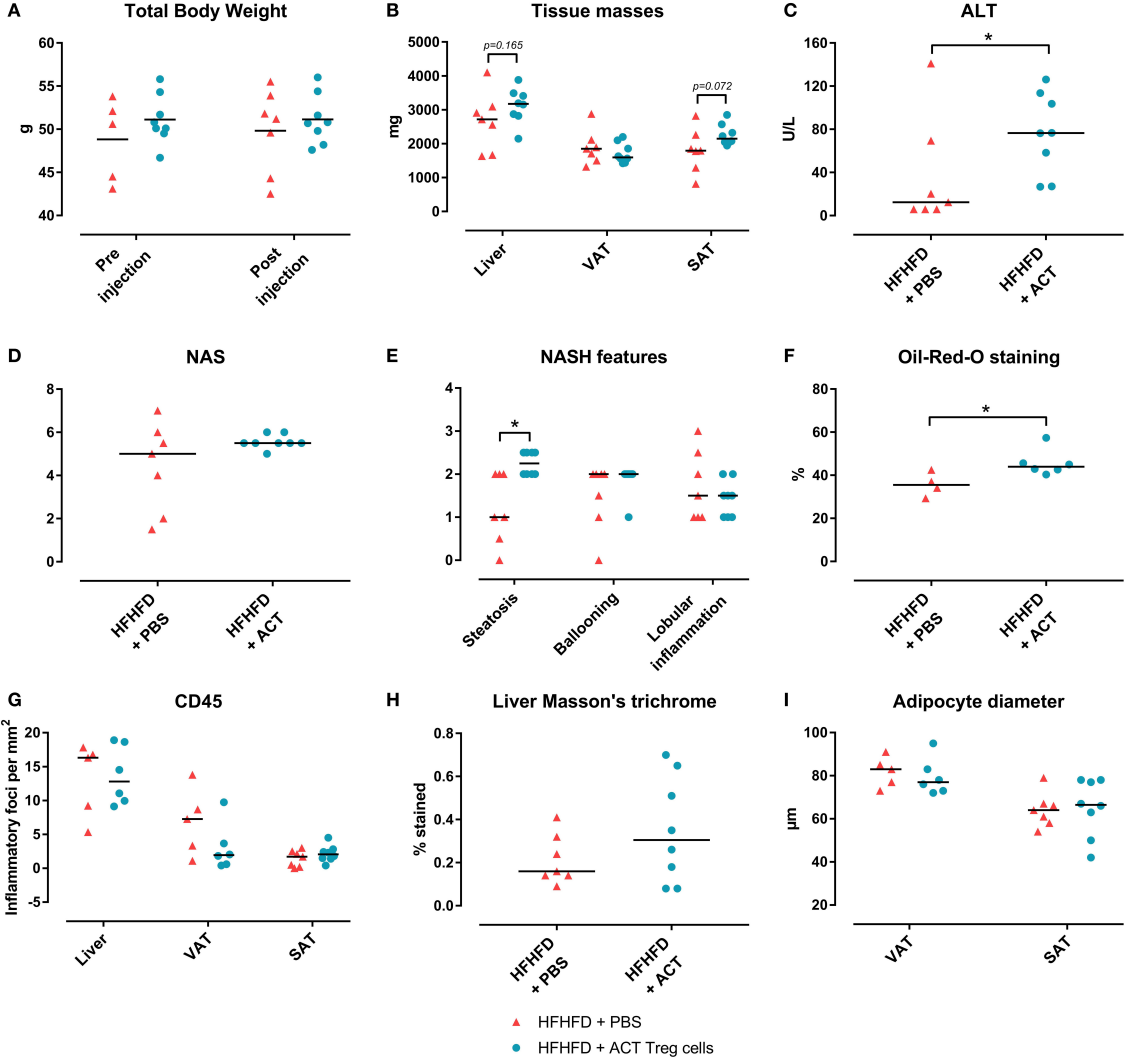
The effect of adoptive Treg cell transfer on metabolic disruptions and NASH. **(A)** Total body weight before and after injection **(B)** Tissue masses. **(C)** Plasma ALT levels. **(D)** NAS. **(E)** NASH features. **(F)** Quantification of hepatic steatosis through Oil-red-O staining, expressed as percentage stained. **(G)** Quantification of hepatic and adipose tissue inflammation through CD45 immunohistochemistry, expressed as number of inflammatory foci per mm^2^. **(H)** Quantification of fibrosis on MT-stained liver tissue, expressed in percentage stained. **(I)** Adipocyte diameter. Medians are depicted with a horizontal black line. *P < 0.05 (Mann Whitney U). ACT, adoptive cell transfer; ALT, alanine transaminase; HFHFD, high-fat high-fructose diet; NAFLD, non-alcoholic fatty liver disease; NAS, NAFLD activity score; NASH, non-alcoholic steatohepatitis; PBS, phosphate-buffered saline; SAT, subcutaneous adipose tissue; Treg, regulatory T; VAT, visceral adipose tissue.

**Figure 4:**
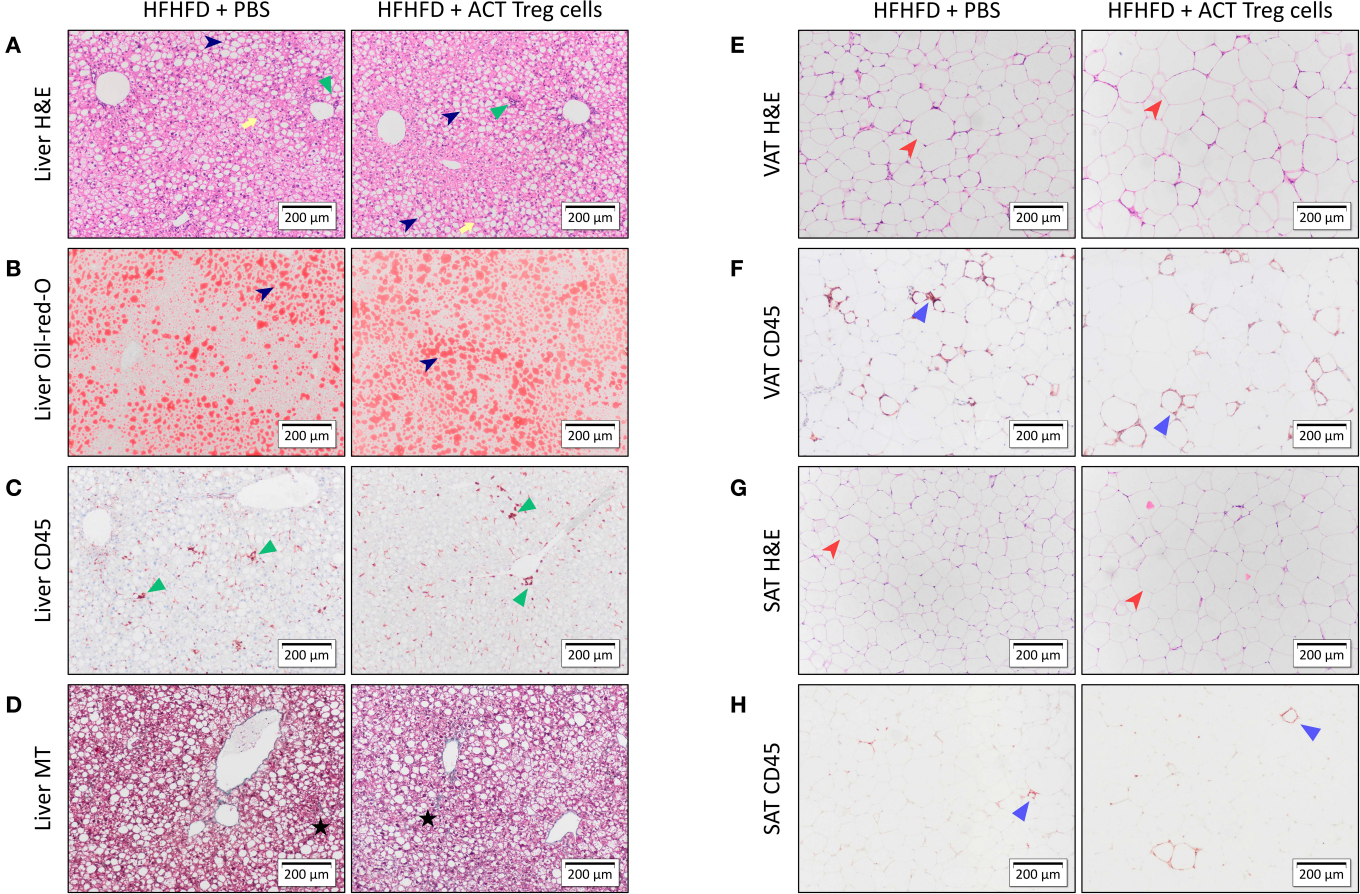
Representative 10x images of liver and adipose tissue histology showing the effect of an ACT of Treg cells on HFHFD-induced NASH. Histological staining was performed on liver tissue with **(A)** H&E, **(B)** Oil-red-O, **(C)** CD45 immunohistochemistry, and **(D)** Masson's trichrome; on VAT with **(E)** H&E and **(F)** CD45 immunohistochemistry; and on SAT with **(G)** H&E and **(H)** CD45 immunohistochemistry. At the level of the liver, the ACT caused an exacerbation of steatosis (dark blue arrowhead), while not affecting hepatic inflammation (green triangle) or hepatic fibrosis (black star). Ballooning is marked with a yellow arrow. At the level of VAT and SAT, no effect of the ACT was observed on adipose tissue diameter (red arrowhead) or adipose tissue inflammation (blue triangle). ACT, adoptive cell transfer; H&E, hematoxylin-eosin; HFHFD, high-fat high-fructose diet; MT, Masson's trichrome; PBS, phosphate-buffered saline; SAT, subcutaneous adipose tissue; Treg, regulatory T; VAT, visceral adipose tissue.

Confirming previously published data, HFHFD feeding induced NASH and multiple metabolic disruptions, which again coincided with a decrease in VAT Treg cell numbers (9). First investigating the effect of the ACT of Treg cells on the proportions of the various T-cell subsets under investigation, unexpectedly, it did not induce an increase in the proportion of Treg cells in the VAT. Conversely, the only investigated tissue that showed an increase in Treg cells was the SAT (Figure 2A). Moreover, a decrease in Th1 cells was observed in all investigated tissues, except for VAT, in which a trend was still present (Figure 2B), whereas no effect was observed on the proportion of Tc or Th17 cells in the investigated tissues (Figures 2C,D). Secondly investigating the effect on the mouse phenotype, the ACT did not induce changes in total body weight (Figure 3A)—nor was a difference in total body weight present before injection—plasma lipid levels, leptin or adiponectin levels (Table 2). However, a trend toward a higher SAT and liver mass was observed (Figure 3B). Furthermore, an increase in ALT levels was shown (Figure 3C). Although histologically no difference was demonstrated in the NAS, nor in the degree of ballooning or lobular inflammation, ACT mice scored higher in steatosis grade (Figures 3D,E, 4A). This was confirmed with automatic quantification of Oil-red-O-stained liver tissue (Figures 3F, 4B). Automated quantification of CD45 immunohistochemistry on liver tissue did not show any differences in hepatic inflammation between the two groups (Figures 3G, 4C). Quantification of MT-stained liver tissue showed no significant difference in fibrosis (Figures 3H, 4D). Lastly, no effect of the ACT was observed on adipocyte diameter or adipose tissue inflammation in VAT as assessed by quantification of CD45 immunohistochemistry, nor in SAT, although the degree of inflammation was low in both groups in this compartment (Figures 3G,I, 4E–H).

**Table 2:** The effect of adoptive Treg cell transfer on biochemical plasma parameters.

**Biochemical plasma analysis**	**Controls**	**ACT Treg cells**	***P***
AST–U/L	198 (128–229)	188 (155–241)	0.779
ALT–U/L	12 (6–45)	77 (51–106)	**0.048**
Total cholesterol–mg/dL	165 (137–171)	178 (159–187)	0.397
LDL–mg/dL	19 (14–21)	19 (16–28)	0.694
HDL–mg/dL	96 (68–106)	105 (89–113)	0.336
Triglycerides–mg/dL	80 (58–90)	64 (58–100)	0.867
Leptin–ng/mL	183 (180–193)	218 (163–256)	0.792
Adiponectin–μg/mL	52 (46–57)	57 (51–63)	0.792

*ACT, adoptive cell transfer; ALT, alanine transaminase; AST, aspartate transaminase; HDL, high-density lipoprotein; LDL, low-density lipoprotein. Significant P values are shown in bold*.

**Figure 5:**
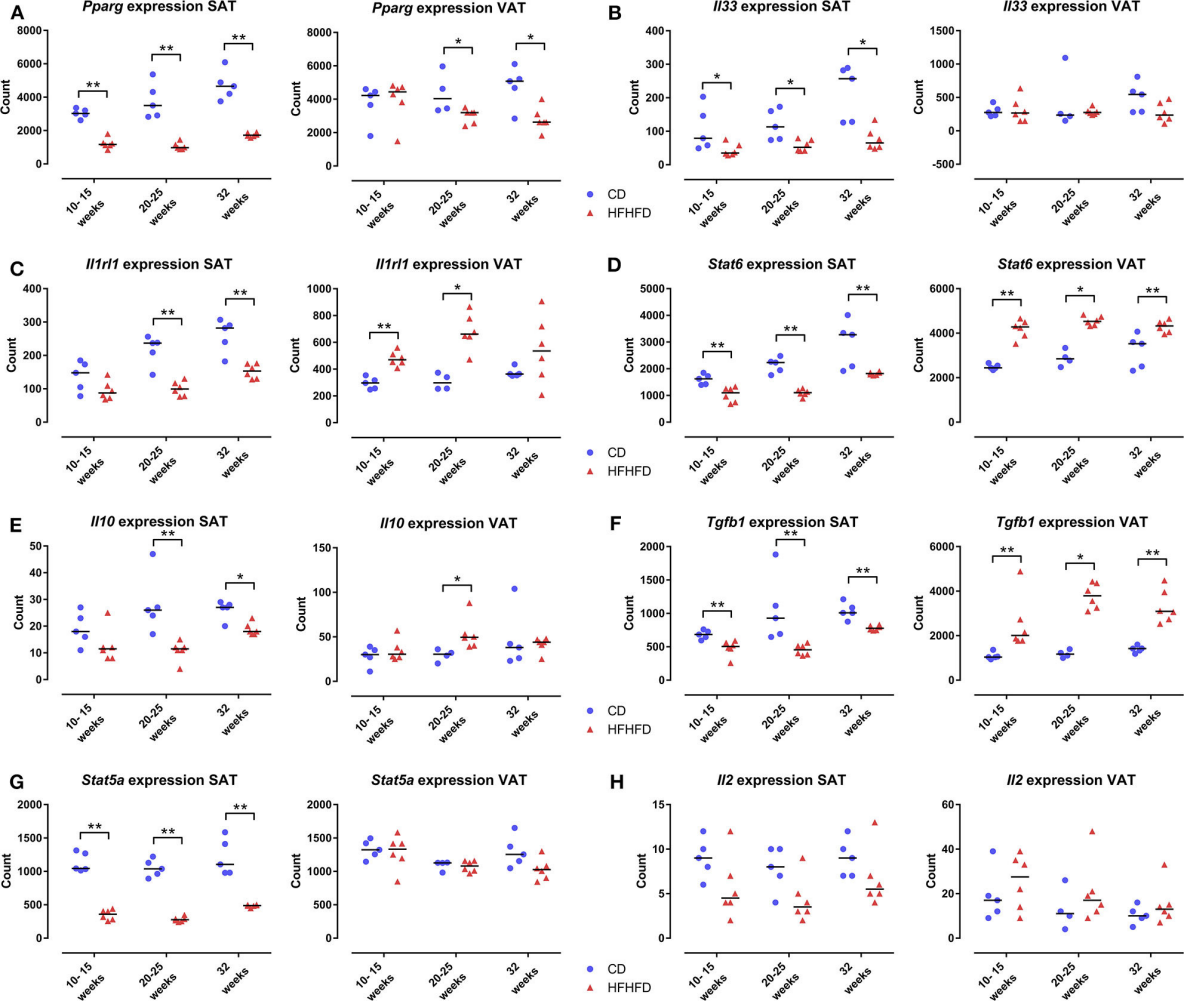
Gene expression in SAT and VAT comparing CD-fed and HFHFD-fed mice at an experimental duration of 10–15, 20–25, and 32 weeks. **(A)** *Pparg*. **(B)** *Il33*. **(C)** *Il1rl1*, coding for the protein ST2. **(D)** *Stat6*. **(E)** *Il10*. **(F)** *Tgfb1*. **(G)** *Stat5a*. **(H)** *Il2*. Gene expression is expressed in normalized counts. Medians are depicted with a horizontal black line. *P < 0.05; **P < 0.01 (Mann Whitney U). CD, control diet; HFHFD, high-fat high-fructose diet; SAT, subcutaneous adipose tissue; VAT, visceral adipose tissue.

As the ACT only increased the proportion of Treg cells at the level of the SAT and not at the level of the VAT, we further explored the mechanism involved. Previously, it has been shown that PPAR-γ is critically involved in Treg-cell accumulation in VAT (5) and that HFHFD feeding results in a downregulation of *Pparg* at the level of the VAT (9). Firstly, we therefore investigated the effect of HFHFD feeding on *Pparg* expression in the SAT and VAT. To do so, we used SAT and VAT samples from mice fed either a CD or HFHFD for 10–32 weeks originating from previous experiments (9). Compared to lean CD-fed mice, obese HFHFD-fed mice showed a significant decrease in *Pparg* expression in the SAT from 10 to 15 weeks, even to a greater degree than was the case at the level of the VAT (Figure 5A). Secondly, recent data support a role for the IL-33/ST2 axis as a positive regulator of Treg cells in the VAT (19). Therefore, we next investigated the effect of HFHFD feeding on *Il33* and *Il1rl1*—the gene encoding ST2—expression in SAT and VAT. HFHFD-fed mice showed a decrease in *Il33* and *Il1rl1* expression compared to CD-fed mice, respectively from 10 to 15 weeks and 20 to 25 weeks, at the level of the SAT, whereas at the level of the VAT, HFHFD feeding increased *Il1rl1* expression at 10–15 and 20–25 weeks, while not affecting *Il33* expression (Figures 5B,C). Thirdly, Kälin et al. have demonstrated a possible role for the STAT6/Pten axis in Treg-cell accumulation specifically in the SAT (20). However, our data show a decrease in *Stat6* expression in the SAT, whereas an increase was observed in the VAT (Figure 5D). Moreover, expression of *Il10, Tgfb1*, and *Stat5a*—other well-known regulators of Treg cell differentiation—was also shown to be downregulated in SAT after HFHFD feeding, whereas no or the opposite effect was observed at the level of the VAT (Figures 5E–G). Lastly, we investigated the expression of *Il2*—another well-known regulator of Treg cell differentiation—but did not find any difference in its expression in HFHFD-fed mice, nor at the level of the SAT or the VAT, although it should be noted that expression levels in both tissue sites were very low (Figure 5H).

## Discussion

We previously demonstrated that HFHFD feeding for 20 weeks induced obesity, hypercholesterolemia, and NASH with increased ALT levels and moderate fibrosis. Moreover, VAT Treg cells were depleted in these HFHFD-fed mice and their numbers were negatively correlated with the severity of NAFLD, as assessed by the NAS (9). We hypothesized that an ACT of Treg cells from healthy donor mice to HFHFD-fed mice would correct this immune disturbance, thereby attenuating the NASH.

Surprisingly, the ACT did not increase the proportion of Treg cells in the VAT, whereas it did so in the SAT. Moreover, the ACT was associated with an increase in ALT levels and hepatic steatosis. Additionally, a decrease in Th1 cells was observed in almost all tissues. This is in contrast to the findings of Ma et al. and Ilan et al. who report a Treg cell-mediated attenuation of hepatic inflammation. However, it should be noted that important differences exist between our study and the work of these authors in both timing and experimental setup, which might have influenced the distribution of the injected cells, as well as the effect on the phenotype. Ma et al. used C57BL/6 mice that were fed a high-fat diet for only 6–8 weeks and were sacrificed 10–12 h after performing an ACT of Treg cells. Additionally, they injected the Treg cells intravenously, whereas we did so intraperitoneally (10). Ilan et al. expanded Treg cell numbers through oral administration of anti-CD3 antibodies and β-glucosylceramide in 8–10 weeks-old ob/ob mice without dietary stimulus (21). In parallel, Eller et al. have previously demonstrated improved insulin sensitivity after repeated intravenous ACT of Treg cells in obese db/db mice (11). On the other hand, the ACT-induced reduction in Th1 cells has been reported by other authors in different models (22, 23) and could be expected because of the immune-controlling effect of Treg cells, although it is somewhat surprising that the same effect was absent at the level of the Th17 cells. Possibly, this potential reduction was obscured by the large degree of plasticity that is known to exist from reg T cells toward Th17 cells and might very well have been present in the environment of low-grade inflammation that is present in the HFHFD-fed mice (4).

Furthermore, it is unclear why the ACT increased the proportion of Treg cells in the SAT, rather than in the VAT, where these cells were shown to be depleted (9). Interestingly, our group previously demonstrated an increase in SAT Treg cells in high-fat diet-fed mice, suggesting a predilection for Treg cell accumulation in the SAT under these conditions (15). This is confirmed in a recent study by Fang et al. that showed a greater accumulation of Treg cells at the level of the SAT than at the level of the VAT after intravenous ACT of Treg cells in db/db mice. Additionally, the authors show that the ACT induced beiging and upregulation of thermogenic genes in the SAT, while not affecting these features at the level of the VAT (24). Similarly, Kälin et al. have demonstrated that CD4^+^ T cells isolated from SAT are more prone to Treg differentiation than CD4^+^ T cells isolated from VAT (20). Moreover, it has been demonstrated in the past that PPAR-γ is critically involved in the accumulation of Treg cells in the VAT (5), while HFHFD feeding was shown to decrease the expression of PPAR-γ in VAT (9). Furthermore, the essential role of PPAR-γ is highlighted by our finding that the expression of other critical genes involved in Treg cell differentiation, including *Il2, Il10, Stat5a, Il33*, and *Il1rl1*, remained largely unaffected by HFHFD feeding at the level of the VAT. Considering all these elements, the HFHFD-induced decrease in *Pparg* expression might therefore explain the lack of an increase in Treg cells in the VAT following the ACT. Conversely, significantly less is known about the mechanisms that drive Treg accumulation in the SAT, including whether or not this involves PPAR-γ (19). However, in this study we have shown that HFHFD feeding resulted in a downregulation of *Pparg* in the SAT, even as it does in VAT, following the same pattern of downregulation as the Treg cell-associated transcription factor *Stat5*. Moreover, we also demonstrated an HFHFD-induced decrease in the expression of many other genes that are involved in Treg cell differentiation, including *Stat6*, which has recently been put forward as a driver of Treg cell differentiation specifically at the level of the SAT (20). It might therefore be possible that Treg-cell accumulation at the level of the SAT is PPAR-γ independent and depends on a yet unknown regulator, resulting in the observed preferred accumulation in this compartment after ACT.

As mentioned above, our group previously demonstrated an increase in SAT Treg cells in mice that were fed a high-fat diet for 36 weeks (15). As this effect was not observed in mice that were fed the HFHFD for 10–32 weeks (9), this presumably constitutes an effect occurring late in the disease process. Possibly, the ACT-induced increase in SAT Treg cells induced an exacerbation of the liver disease parallel to the situation at 36 weeks of high-fat diet feeding.

Alternatively, the detrimental effect of the ACT might be related to the decrease in Th1 cells. Although some human data support an increase in Th1 cells in liver and peripheral blood in NAFLD (25–27) and Rolla et al. report an increase in hepatic Th1 cells in methionine and choline deficient diet-fed mice (28), little is known about the exact effect of these cells on NAFLD. However, they were shown to have an antifibrotic effect, probably in an IFNγ-dependent manner, although no fibrosis-specific data is available in the context of NAFLD (4). One could therefore speculate that the ACT-induced reduction in Th1 cells had an aggravating effect on the HFHFD-induced NASH.

We acknowledge that certain limitations are present in this study. In accordance with an established protocol in our research group (12, 13), we injected the adoptively transfer Treg cells intraperitoneally, whereas the majority of previous studies used intravenous injection. It is possible that the difference in route of administration caused a difference in distribution of the adoptively transferred cells. Moreover, the interval between the ACT and sacrifice was probably not sufficient to expect an effect on fibrosis. To study this feature of NAFLD, repeated ACT of Treg cells is necessary to ensure a long-term effect. Additionally, a model that is more focused on fibrosis and more sensitive fibrotic markers than histology alone should be used when exploring this research goal.

In conclusion, the ACT of Treg cells in HFHFD-fed mice did not lead to the expected attenuation of the metabolic disruptions or the NASH but rather exacerbated the degree of hepatic steatosis. Interestingly, the SAT, rather than the VAT seemed to play a central role in this process, suggesting differential roles for both adipose tissue sites, with the SAT being involved in a later disease stage than the VAT. Moreover, the HFHFD-induced decrease in *Pparg* expression appeared critical in the decrease of Treg cells at the level of the VAT and the inability to replenish the amount of Treg cells by means of an adoptive transfer, while the mechanism of Treg cell accumulation at the level of the SAT remained unclear. Furthermore, the mechanisms behind the exacerbation of steatosis remain unclear, warranting further exploration as they might further unravel the exact alterations in the complex immune responses involved in the pathogenesis of NASH.

## Data Availability Statement

The raw data supporting the conclusions of this article will be made available by the authors, without undue reservation.

## Ethics Statement

The animal study was reviewed and approved by Ethical Committee for Animal Testing, University of Antwerp (Antwerp, Belgium), ID 2015-16.

## Author Contributions

MV conceptualized, designed, and performed the experiments, and wrote the manuscript. LV and SF conceptualized and designed the experiments, supervised the project, and revised the manuscript. WK, TV, DE, PM, JD, LG, and BD provided scientific input and revised the manuscript. All authors contributed to the article and approved the submitted version.

## Conflict of Interest

The authors declare that the research was conducted in the absence of any commercial or financial relationships that could be construed as a potential conflict of interest.
